# A 3D multiscale model of cancer stem cell in tumor development

**DOI:** 10.1186/1752-0509-7-S2-S12

**Published:** 2013-12-17

**Authors:** Fuhai Li, Hua Tan, Jaykrishna Singh, Jian Yang, Xiaofeng Xia, Jiguang Bao, Jinwen Ma, Ming Zhan, Stephen TC Wong

**Affiliations:** 1 NCI Center for Modeling Cancer Development, Department of Systems Medicine and Bioengineering, The Methodist Hospital Research Institute, Weill Medical College of Cornell University, Houston, TX 77030, USA; 2 School of Mathematical Sciences, Beijing Normal University, Laboratory of Mathematics and Complex Systems, Ministry of Education, Beijing 100875, China; 3 Department of Information Science, School of Mathematical Sciences & LMAM, Peking University, Beijing 100871, China

## Abstract

**Background:**

Recent reports indicate that a subgroup of tumor cells named cancer stem cells (CSCs) or tumor initiating cells (TICs) are responsible for tumor initiation, growth and drug resistance. This subgroup of tumor cells has self-renewal capacity and could differentiate into heterogeneous tumor cell populations through asymmetric proliferation. The idea of CSC provides informative insights into tumor initiation, metastasis and treatment. However, the underlying mechanisms of CSCs regulating tumor behaviors are unclear due to the complex cancer system. To study the functions of CSCs in the complex tumor system, a few mathematical modeling studies have been proposed. Whereas, the effect of microenvironment (mE) factors, the behaviors of CSCs, progenitor tumor cells (PCs) and differentiated tumor cells (TCs), and the impact of CSC fraction and signaling heterogeneity, are not adequately explored yet.

**Methods:**

In this study, a novel 3D multi-scale mathematical modeling is proposed to investigate the behaviors of CSCsin tumor progressions. The model integrates CSCs, PCs, and TCs together with a few essential mE factors. With this model, we simulated and investigated the tumor development and drug response under different CSC content and heterogeneity.

**Results:**

The simulation results shown that the fraction of CSCs plays a critical role in driving the tumor progression and drug resistance. It is also showed that the pure chemo-drug treatment was not a successful treatment, as it resulted in a significant increase of the CSC fraction. It further shown that the self-renew heterogeneity of the initial CSC population is a cause of the heterogeneity of the derived tumors in terms of the CSC fraction and response to drug treatments.

**Conclusions:**

The proposed 3D multi-scale model provides a new tool for investigating the behaviors of CSC in CSC-initiated tumors, which enables scientists to investigate and generate testable hypotheses about CSCs in tumor development and drug response under different microenvironments and drug perturbations.

## Background

The mechanisms of tumor initiation, progression, metastasis and drug resistance remain elusive due to the complex system of tumors. Recent studies have shown that a sub-population of tumor cells, named cancer stem cells (CSCs) or tumor initiating cells (TICs) tumor are responsible for tumor development and drug resistance [1-3]. The CSC concept is still controversial, as it is difficult to discover and validate cancer stem cells, particularly their unlimited self-renewal and differentiation capabilities [1]. However, CSCs have been being isolated from more and more cancers [2], since first discovered in the acute myeloid leukemia (AML) by using CD34^++^/CD38^- ^biomarkers [3]. Recently, the breast CSCS were identified by using CD44^+^CD24^-/low ^biomarkers in [4], and the colon CSCS were also reported [5]. CSCs are believed to have strong self-renewal capacity, could differentiate into heterogeneous tumor populations through asymmetric division [6,7], and are responsible for drug resistance and metastasis [8,9]. Reportedly, CSCs are heterogeneous with different self-renewal and tumor formation abilities, which might be caused by varying activation intracellular signaling (e.g., Wnt, Shh and Stat3) due to the diverse concentrations of external mE factors [10,11].

However the roles of CSCs in tumor development remain unknown because of tumor complexity in multiple levels, including signaling transduction, cell-cell communication, and cell-microenvironment interactions. The mathematical simulation models have been powerful tools for understanding the tumor systems [12]. In general, the existing mathematical models of the tumor development can be grouped into three major categories: discrete, continuous and hybrid. The discrete models, e.g., cellular automata [13] and Glazier and Graner model [14], simulate cell behaviors individually with a group of rules. The continuous models employ ordinary or partial differential equations to simulate the behaviors of tumor cell populations and dynamics of mE factors [15,16]. The hybrid models are the combination of the discrete (for modeling cells) and continuous (for modeling mE factors) models [12]. A few mathematical simulation studies have been developed to study CSCs functions in tumor development [17-20]. Whereas, the interactions between mE factors and CSCs, PCs and TCs, the impact of the heterogeneity and fraction of CSCs, have not been adequately considered in the mathematical modeling.

**Figure 1:**
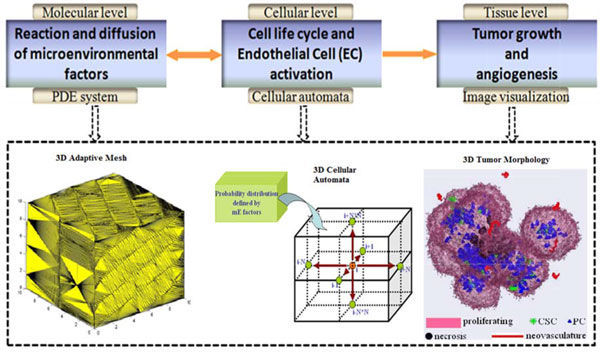
**The schematic of the 3D multiscale model and the implementation**. The molecular scale describing the diffusion and reaction processes of mE factors with PDEs. The cellular level models the behaviours of cells with a 3D cellular automata. The tissue level visualizes the whole tumor morphology.

**Figure 2:**
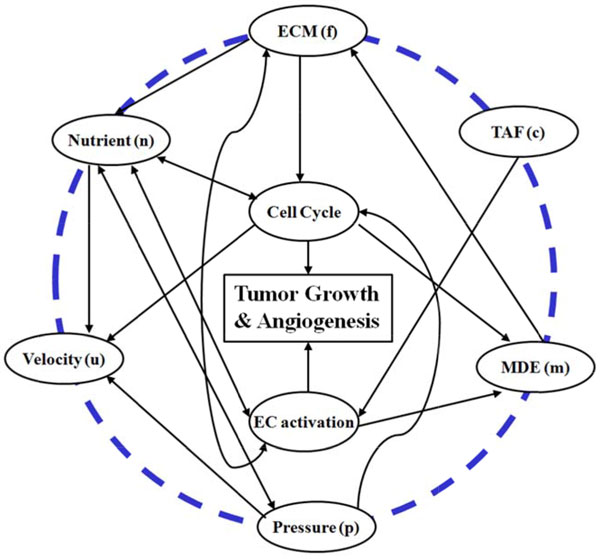
**The interactions between micro-environmental (mE) factors and tumor cells**. The outer circle stands for mE factors being considered at the molecular level, the inner circle corresponds to the processes investigated at the cellular level. The interrelations are denoted by the solid arrows.

Herein, a 3D multi-scale mathematical modeling is proposed to study the functions of CSCs in tumor development. The model, extended our previous concept [21], enables us to study the roles of CSCs in tumor progression and chemo-drug resistance by simulating the tumor growth initiated by a set of heterogeneous CSC populations. The overview of the proposed multi-scale model is shown in Figure 1. In brief, mE factors are considered, including nutrients (e.g., oxygen and glucose), tumor angiogenesis factors (TAF), matrix degrading proteolytic enzyme (MDE), extracellular matrix (ECM), tissue pressure [22], and motility of cells are described in the molecular scale. The interactions between mE factors and cancer cells are shown in Figure 2. The behaviors of endothelial and cancer cells are described by the cellular automata in the cellular scale. The cancer cells are represented by a hierarchical organization of cellular subtypes [23,24], including CSC, a series of intermediate PCs and TCs, each endowed with different biological traits. At the tissue level, the tumor volume, shape and spatial distributions of tumor cells are investigated.

## Methods

### The PDE system characterizing reaction-diffusion process of mE factors

Five mE factors are considered in this model, including nutrients (*n*), tumor angiogenic factor (TAF) (*c*), matrix degrading proteolytic enzyme (MDE) (*m*), extracellular matrix (ECM) (*f*), and tissue pressure (*p*). A system of PDEs is used to delineate the diffusion and reactions mE factors. The *χ_Ω _*is defined as follows.

$$\chi_{\Omega }\left(x\right)=\left\{\begin{array}{c} 1\begin{array}{cc} , & x\in \Omega \end{array}\\ 0\begin{array}{cc} , & x\notin \Omega \end{array} \end{array}\right)$$

The diffusion and reaction of nutrients are modeled by the quasi-steady equation with non-zero Dirichlet boundary conditions [25,26]. Since they are much smaller comparing to cells, nutrient molecules diffuse quickly through ECM at each time point.

(1) $$\left\{\begin{array}{l} 0=\overset{diffusion}{\overset{︷}{D_{n}\nabla^{2}n}}+\overset{production}{\overset{︷}{\chi_{\left(\Omega_{V}\cup \Omega_{H}\right)}\left(1-n\right)\left(\lambda_{pa}^{n}\left(1-p\right)\chi_{\sum_{C}}+\lambda_{pp}^{n}\right)+\lambda_{b}^{n}f}}-\overset{uptake}{\overset{︷}{\lambda_{u}^{n}n}}\\ n{|}_{\partial \Omega }=1 \end{array}\right)$$

where *D_n _*is the diffusion rate of nutrient molecules, and $\lambda_{pp}^{n}$, $\lambda_{pa}^{n}$ denote the nutrient molecules transferring rates from pre-existing and neo-vasculature vessels. The $\lambda_{b}^{n}$ is the nutrient molecule binding rate to fibronectin; $\lambda_{u}^{n}$ is the uptake rate by cells, and it is different for specific types of cells. The χ_Σc _is an indicator function that equals to 1 at the new generated vessels. The term (1-*p*) is used to indicate the difference of nutrient molecule transfer with different pressure, and (1-*n*) is to reflect nutrient molecules' saturation effect.

The TAF is selected by tumor cells, and during its diffusion, TAF will be either degrade naturally or captured by endothelial cells. This process is described as follows:

(2) $$\left\{\begin{array}{l} 0=\overset{diffusion}{\overset{︷}{D_{c}\nabla^{2}c}}+\overset{production}{\overset{︷}{\lambda_{pN}^{c}\chi_{\partial \Omega_{N}}+\lambda_{pV}^{c}\chi_{\Omega_{V}}}}-\overset{uptake/degradation}{\overset{︷}{\lambda_{u}^{c}c\chi_{\sum_{c}}-\lambda_{d}^{c}c}}\\ \frac{\partial c}{\partial \vec{n}}{|}_{\partial \Omega }=0 \end{array}\right)$$

where *D_c _*is the diffusion rate of TAF, $\partial \Omega_{N}$ is the boundary of necrotic and viable regions, the $\vec{n}$ is the unit outer normal direction, $\lambda_{pN}^{c}$, $\lambda_{pV}^{c}$ are the TAF secretion rates by dying and viable tumor cells, respectively; $\lambda_{u}^{c}$ is the uptake rate by endothelial cells, and $\lambda_{d}^{c}$ is the rate of natural degradation.

The ECM, such as fibronectin, represents a set of binding molecules that do not diffuse but increase the tumor cell adhesion. The concentration of ECM molecules is measured as follows:

(3) $$\left\{\begin{array}{l} \frac{\partial f}{\partial t}=\overset{production}{\overset{︷}{\lambda_{p}^{f}\left(1-f\right)\chi_{\Omega_{V}}+\lambda_{sp}^{f}\chi_{\Sigma_{C}}}}-\overset{degradation}{\overset{︷}{\lambda_{d}^{f}fm}}\\ f{|}_{t=0}=1 \end{array}\right)$$

Where the $\lambda_{p}^{f}$, $\lambda_{sp}^{f}$ denote fibronectin production rates by tumor and endothelial cells, respectively, and $\lambda_{d}^{f}$ is the ECM degradation rate by MDE.

MDE is secreted by endothelial cells and viable tumor cells to degrade ECM. The MDE diffusion and reaction processes are defined as:

(4) $$\left\{\begin{array}{l} 0=\overset{diffusion}{\overset{︷}{D_{m}\nabla^{2}m}}+\overset{production}{\overset{︷}{\lambda_{p}^{m}\left(1-m\right)\chi_{\Omega_{V}}+\lambda_{sp}^{m}\chi_{\Sigma_{C}}}}-\overset{decay}{\overset{︷}{\lambda_{d}^{m}m}}\\ \frac{\partial m}{\partial \vec{n}}{|}_{\partial \Omega }=0 \end{array}\right)$$

In this equation, *D_m _*is the diffusion coefficient, $\lambda_{p}^{m}$ and $\lambda_{sp}^{m}$ denote MDE secretion rates by the endothelial cells and viable tumor cells, and $\lambda_{d}^{m}$ is the MDE decay rate.

The cell velocity is resulted from the different cell proliferation at different regions, and is described by using the following Darcy-Stokes law [27].

(5) $$\vec{u}=-\nabla p\begin{array}{cc} , & p{|}_{\partial \Omega }=0 \end{array}$$

And the velocity field follows the divergence equation:

(6) $$\nabla \cdot \vec{u}=\chi_{\Omega_{V}}\left(n-\lambda_{a}\right)-\chi_{\Omega_{N}}\lambda_{N}$$

where, *λa *and *λ_N _*are the volume loss rates caused by cellular apoptosis. The first term on the right of equation (6) is the source effect, while the second term can be considered as the sink effect. The diffusion of pressure is obtained by taking the divergence operation on both sides of equation (5), and combined with equation (6):

(7) $$\left\{\begin{array}{l} 0=\nabla^{2}p+\left(n-\lambda_{a}\right)\chi_{\Omega_{V}}-\lambda_{N}\chi_{\Omega_{N}}\\ p{|}_{\partial \Omega }=0 \end{array}\right)$$

In the implementation, the finite element method is used to solve PDEs with diffusion item $\nabla^{2}$[28]. The ECM equation is solved by the 2^nd ^order total variation Runge-Kutta method [29]. The time interval, Δt, is calculated to keep the stability of the PDEs:

(8) $$\Delta t=\frac{\Delta l}{4}\text{min}\left\{\frac{1}{{\text{max}}_{i}|V_{i}|},\frac{1}{{\text{max}}_{i}|\vec{u_{i}}|}\right\}$$

where Δl=0.1 is the spatial interval, $V_{i}$ is the function defined on the TAF, ECM, and cell velocity in [25], and $\vec{u_{i}}$ is the cell velocity at *i*-th spatial point [25].

### 3D Cellular Automata

**Figure 3:**
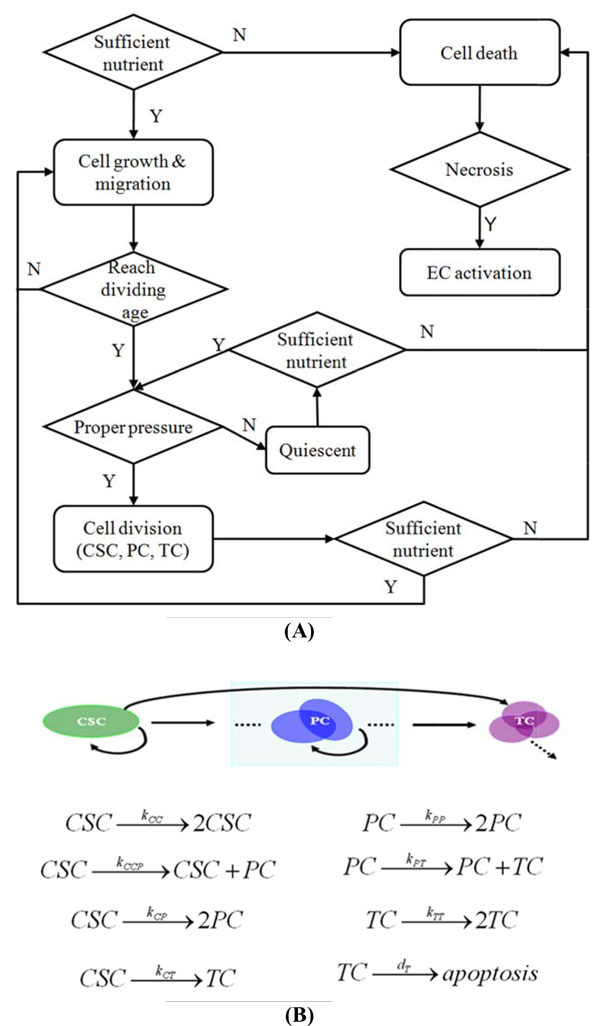
**The flowchart of control in the multiscale model of CSC-initiated tumor development**. (A) Each cell evolves according to their life cycle under the conditions confined by the PDE system. Necrosis occurs when cell death is induced by hypoxia. (B) Hierarchical organization of different cell subtypes and their proliferative kinetics. Cancer stem cells (CSC) expand their own population by symmetric proliferation to two identical daughter cells still with CSC-like traits and expand the whole tumor through asymmetric differentiation to progenitor cells (PC) and terminally differentiated cells (TC) in this model. Similarly, PCs contribute to the constitution of the tumor in a similar way, but do not reversibly produce CSCs according to the hierarchical organization hypothesis. The TCs are assumed to either proliferate or be apoptotic at each time point without ability to divide into any other subtypes. The parameters above the arrows indicate the occurrence probability of the referred event at each time point.

For simplicity, cells are limited to interact with its six immediate orthogonal neighbor grids such that the cell can move or proliferate, based on probabilities calculated from the distribution of surrounding mE factors. Figure 3 shows a flowchart of cell behavior under interactions with mE factors and cell lineage in proliferation. Specifically, for a cell being at location *k*:

1) Check available (empty) neighbor locations.

2) If there is no available neighbor location, go to step 8).

3) Calculate the motion probabilities to the *m (m <= 6) *available neighbor locations as: *q_i _= n_i_/f_i_, i = 0, 1, ..., m*, where *q_0 _*means the probability of staying at the same location.

4) Denote $q_{k}^{\prime }=\sum_{i=1}^{k}q_{i}$, and normalize them as: $q_{i}^{\prime \prime }=q_{i}^{\prime }/q_{m}^{\prime }$.

6) Define $R_{1}=\left[0,q_{1}^{\prime \prime }\right]$, and $R_{i}=\left[q_{i-1}^{\prime },q_{i}^{\prime \prime }\right]$, *i = 1, 2, ..., m*.

7) Pick up a number *r *randomly from [0, 1], then move the cell to the *i*-the neighbor location where r belongs to.

8) Update the cell age, and then check if the cell is mature to divide. If yes, add one new cell with right cell type to a neighbor location with the above rules. Check the mE conditions to determine if the cell should enter the quiescent or death status.

### Simulation of chemotherapy

In simulation of chemotherapy, all cancer cells (CSCs, PCs, TCs) can be killed by chemo drugs with different doses. Since the diffusion and reaction processes of the drug molecules resemble that of nutrients, we use the same diffusion-reaction equation for drugs. We assume that TCs proliferation is reduced due to the effects of drugs, while CSC proliferation is accelerated as activated by the volume loss due to the drug effect, which is parallel to the normal stem cell functions [30]. We adjust the proliferation age of each cell subtype in such a way to stimulate the reaction of tumor cells to chemotherapy.

### The Gompertz curve fitting

The tumor growth pattern is fitted by using the Gompertz curve, which shows slow change in the beginning and the end of tumor growth [31]. Though for tumors that undergo angiogenesis, a plateau in growth is not necessarily reached, the Gompertz cure is an appropriate choice in modeling the initial phase of the tumor growth with limited access to nutrients. Mathematically the tumor growth is represented as:

(9) $$ŷ\left(t\right)=y_{0}e^{k\left(1-e^{-b\left(t-t_{0}\right)}\right)}$$

where $y_{0}$ is the volume of tumor at time $t_{0}$, while positive constants *k*, *b *denote the axis displacement and growth speed. The least square is used to determine the optimal parameters as follows:

(10) $$\left(\overset{\text{\_}}{k},\overset{\text{\_}}{b},\overset{\text{\_}}{y_{0}},\overset{\text{\_}}{t_{0}}\right)=\underset{\left(k,b,y_{0},t_{0}\right)}{\text{arg}\;\text{min}}\overset{N}{\underset{i=1}{\sum }}{\left(ŷ\left(t_{i}\right)-y_{i}\right)}^{2}$$

Here, ŷ(t) is the tumor volume growth function defined in equation (9), $y_{i}$ is the measured volume of tumor at time point *i*, obtained either from biological experiments or by computer simulation, and *N *is the number of available measurements.

### Measurements of tumor development

The tumor properties are evaluated by following measurements: proliferation potential (*PP*), time to reach potential (*TtP*), average aggressive index (*AAI*), and average fitting error (*AFE*). *PP *is defined as (9):

(11) $$PP=\underset{t\to +\infty }{\text{lim}}ŷ\left(t\right)=y_{0}e^{k}$$

The *PP *value could not predict the final volume of a tumor because of many unforeseen contributing factors when tumors grow large. However, it can be used to compare the potential volume of tumors in a relative sense. *TtP *is estimated by solving an inverse problem of (9), that is, by searching the time when ŷ(t) reaches *PP *for the first time:

(12) $$TtP=\text{inf}\left\{t>0:ŷ\left(t\right)\geq PP\right\}$$

where '*inf*' is the *infimum *operation. *AAI *is represented as:

(13) $$AAI=\frac{1}{N}\overset{N}{\underset{i=1}{\sum }}\frac{S_{surface}^{i}}{V_{tumor}^{i}}$$

Here, $S_{surface}^{i}$ and $V_{tumor}^{i}$ stand for the surface area and volume of the tumor at time point *i*, which are represented by the number of cells on the surface and the total number of cells composing the tumor respectively. The performance of the curve fitting is assessed by AFE, estimated by the least square method for (10):

(14) $$AFE=\sqrt{f_{obj}/N}$$

where $f_{obj}=\overset{N}{\underset{i=1}{\sum }}{\left(ŷ\left(t_{i}\right)-y_{i}\right)}^{2}$.

## Results

### Simulation of tumor development under different CSCs contents

**Figure 4:**
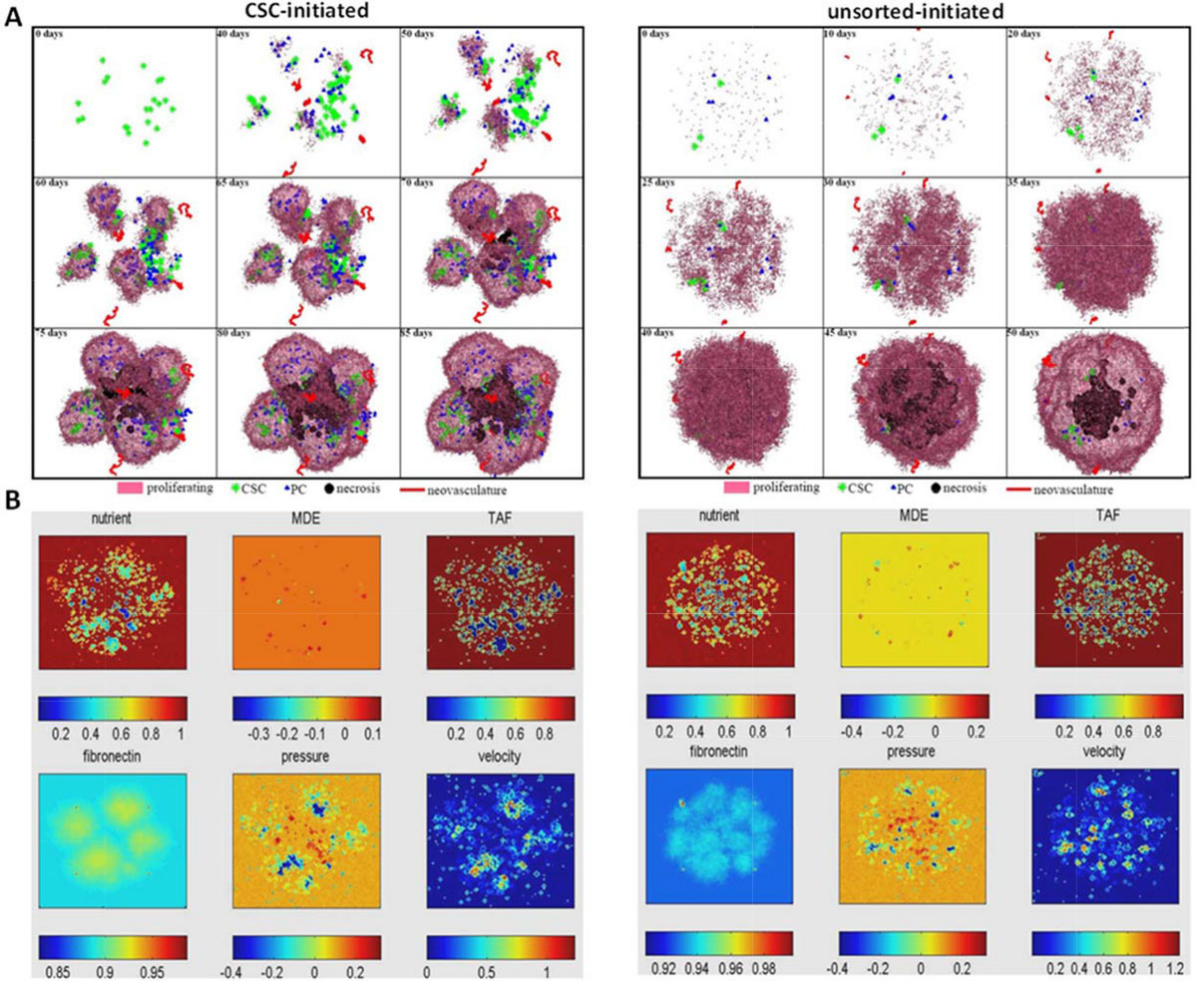
**Simulation of tumor development under different CSC contents**. (A) Time evolution of tumors initiated by pure CSCs and unsorted tumor cells. (B) The corresponding concentration profiles of micro-environmental (mE) factors.

**Figure 5:**
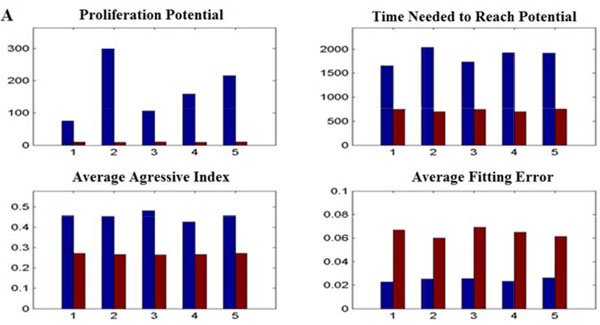
**Quantitative comparison between tumor development initiated from pure CSCs and unsorted tumor cells**. The proliferation potential, time needed to reach potential, average aggressive index, and average fitting error of tumors are compared between two kinds of tumors in five simulations.

To investigate tumor development initiated from a set of tumor cells with different CSC contents (or fractions), herein, we simulated tumor growth with pure CSCs (CSCs only, initiated from ~20 CSCs) and unsorted (mixtures of CSCs and non-CSCs) tumor cells (200 tumor cells in which 4% are CSCs), respectively. Figure 4 shows the dynamics of tumor growth and concentration profiles of mE factors over the time under different CSC contents. As shown, the tumor initiated from the mixed tumor cells grows faster than that tumors initiated from pure CSCs. Whereas the tumors initiated from pure CSCs have more spiky morphology. This might be caused by biased migration of CSCs toward locations (without the limitation of other tumor cells) with a higher nutrient concentration and lower ECM concentration, which affect the tumor geometric morphology. We conducted growth curve fitting of the simulation results and found that the tumor initiated from pure CSCs has an elevated proliferation potential, though it takes a longer time to grow to its limit size (Figure 5). Tumors also exhibits a stronger aggressiveness compared to tumors initiated from the mixed tumor cells (Figure 5).

### Important parameters to tumor growth

**Figure 6:**
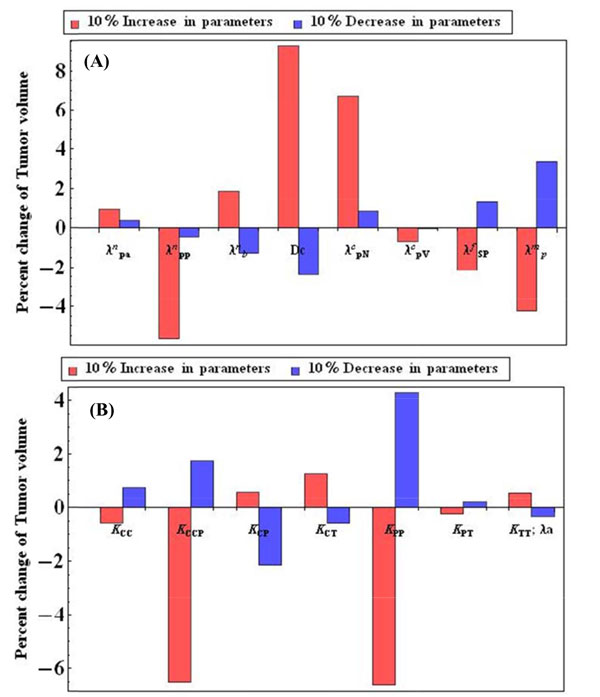
**Sensitivity analysis (A) The sensitivity analysis of the continuous parameters**. (B) The sensitivity analysis of the proliferation related parameters.

In Table 1, all parameters of the proposed multi-scale model of tumor growth are listed. These parameters are determined mostly from either literature or experimental data. We performed sensitivity analysis to discover important parameters to tumor growth initiated from pure CSCs. The parameter values were perturbed in a range of 10%. Figure 6 shows the effects of parameters on the tumor growth. Some diffusion reaction related parameters, e.g., $\lambda_{pp}^{n}$, $\lambda_{pN}^{c}$, $\lambda_{p}^{m}$, and D_c _are sensitive to the tumor growth, and some parameters proliferation abilities parameters, i.e., K_CCP _and K_PP _are also sensitive to tumor growth.

**Table 1:** Model parameters.

Parameter Symbol	Parameter Annotation	Parameter Value	Reference
*D_n_*	Diffusion rate of Nutrient	1.0	P. Macklin et al. (2009)

*D_c_*	Diffusion rate of TAF	100	Estimated

*D_m_*	Diffusion rate of MDE	1.0	P. Macklin et al. (2009)

$\lambda_{u}^{n}$	Uptake rate of Nutrient	[0.2, 0.5, 0.33, 0.67, 1, 1]	X. Zheng et al. (2005); Estimated

$\lambda_{b}^{n}$	Binding rate of nutrient	2.5·e-3	Estimated

$\lambda_{pa}^{n}$	Nutrient transfer rate from neo-vasculature	0.05	X. Zheng et al. (2005); Estimated

$\lambda_{pp}^{n}$	Nutrient transfer rate from existing vessel	0.01	Estimated

$\lambda_{pN}^{c}$	TAF secretion rate by dying cells	0.05	Estimated

$\lambda_{pV}^{c}$	TAF secretion rate by viable cells	0.004	Estimated

$\lambda_{d}^{c}$	TAF degradation rate	0.01	P. Macklin et al. (2009)

$\lambda_{u}^{c}$	TAF uptake rate by endothelial cells	0.025	P. Macklin et al. (2009)

$\lambda_{p}^{m}$	MDE secretion rate by viable cells	{50, 100, 150}	P. Macklin et al. (2009); Estimated

$\lambda_{sp}^{m}$	MDE secretion rate by endothelial cells	1.0	P. Macklin et al. (2009)

$\lambda_{d}^{m}$	MDE degradation rate	10	P. Macklin et al. (2009)

$\lambda_{p}^{f}$	ECM secretion rate by viable cells	0.1	P. Macklin et al. (2009)

$\lambda_{sp}^{f}$	ECM secretion rate by endothelial cells	0.01	Estimated

$\lambda_{d}^{f}$	ECM degradation rate	0.01	P. Macklin et al. (2009)

*λ_a_*	Volume loss rate due to apoptosis	0~0.00013	Estimated

*λ_N_*	Volume loss rate due to necrosis	0.25	X. Zheng et al. (2005)

*θ_a_*	Nutrient dose for cell survival	{0.1, 0.17, 0.25}	X. Zheng et al. (2005); Estimated

*θ_d_*	Maximum drug concentration for cell survival	{0.25, 0.27, 0.375}	Estimated

[*K_CC_*, *K_CCP_*, *K_CP_*, *K_CT_*]	CSC proliferation probabilities	{0.6, 0.25, 0.1, 0.05}	Estimated

[*K_PP_*, *K_PT_*]	PC proliferation probabilities	{0.25, 0.75}	Estimated

[*K_TT_*, *d_T_*]	TC proliferation probability	[1- *λ_a_*, *λ_a_*]	Estimated

*A_p_*	Relative proliferation ages	[1, 0.4, 1, 0.2]	Estimated

*G_m_*	Maximum generations a cell can divide	[250, 50, 25]	Estimated

*C_s_*	Constant for cell size scaling	10·e-5	Estimated

NOTE:

P. Macklin et al. (2009): Macklin, P., et al., *Multiscale modelling and nonlinear simulation of vascular tumour growth*. J Math Biol, 2009. **58**(4-5): p. 765-98.

X. Zheng et al. (2005): Zheng, X., S.M. Wise, and V. Cristini, *Nonlinear simulation of tumor necrosis, neo-vascularization and tissue invasion via an adaptive finite-element/level-set method*. Bull Math Biol, 2005. **67**(2): p. 211-59.

### Tumor response to drug treatment

**Figure 7:**
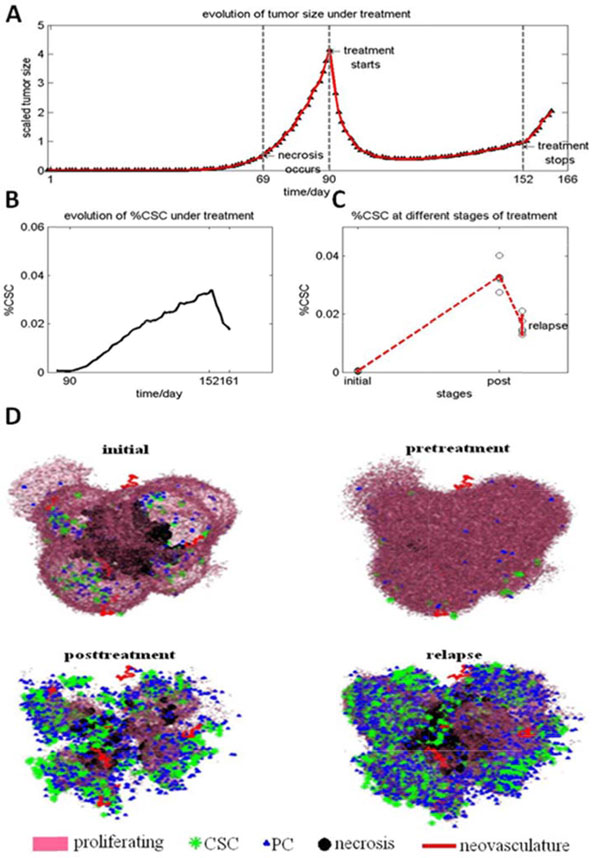
**Simulation of chemo-drug therapy response**. (A) A 9-week treatment to a tumor initiated from pure CSCs. The tumor volume first reduces fast, then remains stable during drug treatment; whereas it grows back quickly after the treatment stops. (B) The evolution of the CSC fraction in the process. (C) The 3D morphology of the tumor at different stages of the treatment.

The effect of chemo-drug treatment on tumor development and CSC fraction is also investigated. Figure 7 shows the dynamics of tumor development under chemo-drug treatment. As can be seen, the solid tumor shrinks during the drug treatment, whereas the CSC fraction increases. The simulation results also reveal that tumors will grow fast to half of its original volume after stop the drug treatment. This fast relapse may be because some CSCs reside in the interior of a tumor where drug molecules are not accessible. It might be due to that the fast proliferating non-CSCs dominate the outer rim of a tumor, and drug molecules will first kill the non-CSCs in order to reach the area where CSCs tend to gather. The quick relapse of tumor, once the treatment stops, as observed in our simulations, can be explained by the escape of CSCs from therapeutic interventions. The decreased percentage and lower proliferation rate of CSCs may be because these interior CSCs reside in a region where the access to nutrients is limited.

### Simulation of CSC self-renewal heterogeneity in tumor development

**Figure 8:**
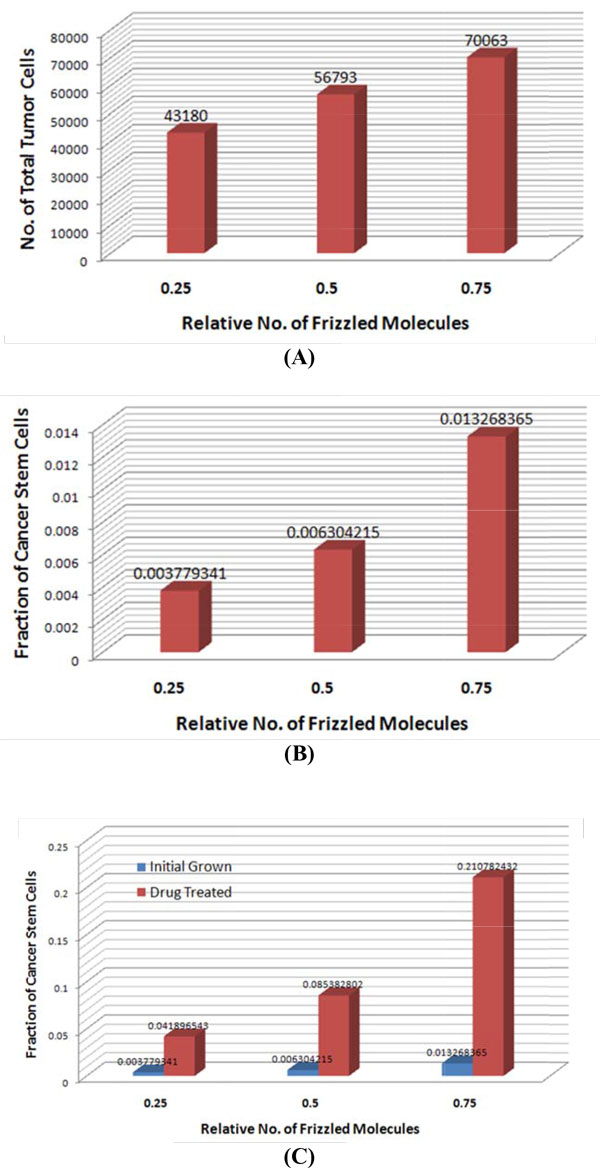
**Comparison of tumor development initiated from single CSCs with heterogeneous self-renewal ability**. (A) Average size of tumors and (B) average CSC fractions of tumors initiated from a single CSC with relative low (0.25), medium (0.5) and high (0.75)self-renewal ability. (C) Average CSC fractions of tumors before and after chemo-drug treatment.

To investigate the heterogeneity of CSC self-renewal ability, we further extend our model as follows: 1) a tumor is initiated from a single CSC; 2) the initiating CSCs have different self-renewal abilities that are proportional to the Gaussian distribution with different means: p∝G(μ,σ), where G(μ,σ) is a Gaussian with mean *μ *and variance $\sigma^{2}$; and 3) the newly formed CSCs during tumor development will obtain a mean value randomly sampled from the $G\left(\mu_{p},\sigma \right)$, where $\mu_{p}$ is the mean of its parent CSC. In our simulation, we set μ=0.25, 0.5, and 0.75, respectively, to indicate the relative low, medium, and high self-renewal ability of initiating CSCs (with fixed σ=0.1). The simulation starts from a single CSC to 30 days, then the same chemo-drug treatment is applied. The virtual chemo-drug treatment will be stopped when 85% of tumor cells are killed. Figure 8 shows the simulation results for the size and fraction of CSCs of tumors initiated from CSCs with different self-renewal abilities. As shown, before chemo-drug treatment, the tumor initiated from CSCs with high self-renewal ability has an average bigger tumor volume (about 1.5 fold), whereas the difference of the CSC fraction is about 2 fold. After chemo-drug treatment, the difference in the CSC fraction is significantly increased to about 3 fold, though the CSC fractions of all tumors are increased significantly comparing to those before chemo-drug treatment. The results indicated that though the pure chemo-drug treatment could reduce the size of tumor, it might also increase the aggressiveness of tumor due to the increased fraction of CSCs, especially for the tumor with the CSCs that have high self-renewal ability. Therefore, the combinations of chemo-drugs and anti-CSCs drugs are needed to achieve better treatment outcomes.

## Discussion & conclusions

Here, a multi-scale and multi-factorial computational model is established in 3D space to study the behaviors and roles of CSCs in leading tumor development. The model is implemented at three hierarchical scales (molecular, cellular, tissue scales). The molecular subsystem characterizes the diffusion and reaction processes of mE factors by using PDEs. The cellular level subsystem simulates the proliferation and migration of all cancer cells and endothelial cells, considering the availability of mE factors, with a 3D cellular automaton. The tissue level subsystem evaluates the temporal and spatial variations of tumor morphology by, four indices. The model can be conveniently expanded to a particular application to generate testable hypotheses.

The simulation studies based on the multi-scale model could provide important insights into tumor development and treatment. For example, the simulation indicated that tumors in mice model initiated by the sorted CSC population had stronger aggressiveness and proliferation potential comparing to tumors from unsorted cancer cells. Also the simulation demonstrated that the neo-vasculature could grow into the interior of a tumor, suggesting a possibility of delivering drugs via neo-vasculature to target the CSCs in the interior of tumors, besides the anti-angiogenic therapy that elicits increased local invasion and distant metastasis of tumors [32,33]. In addition, the simulation indicated that pure chemo-drug treatment may increase the fraction of CSCs significantly, especially for the tumor with CSCs of high self-renewal ability, and consequently, the tumor residual will be more chemo-resistant and aggressive. Thus, a combination of chemo-drugs with CSC inhibition drugs would be more effective in cancer treatment without increasing tumor drug-resistance and aggressiveness.

Many parameters in our model were defined based on general understanding of tumor development through literature mining in this study, as in many other modeling studies. Despite limited experimental data used in defining the parameters, the model we proposed is still valid. It enables us to identify important CSCs behavior and interactions with interested mE factors, and to virtually test hypotheses that cannot be done in an animal model. The simulation studies based on the model can lead to new insight of CSCs in tumor development and shed light on the treatment. As more experimental data become available through our studies, the parameters can be better defined and calibrated, and the resulting model will be better predictable.

Several improvements of the proposed 3D tumor growth model will be conducted in the future work. Cancer is a complex and heterogeneous disease. CSCs from different types of cancer might have different functions and regulatory signaling pathways. With more data of cancer-specific signaling pathways, cell differentiation lineages, stromal cells, and detailed cell-cell interactions becoming available, the proposed model could be extended to study the CSCs in specific cancer types. Also the cell shape could be taken into account as it plays an important role in interactions between cells and mE factors, particularly when the cell density is high and causes shape deformation and cell-cell interaction through cell surface markers. On the other hand, relevant regulatory or signaling pathways could be integrated to refine the modeling. In addition, specific drug effects on different cell cycles and cell types could be considered.

## Competing interests

The authors declare that they have no competing interests.

## Authors' contributions

FL, MZ, SW conceived the study. FL, TH, MZ designed and coordinated the study and drafted the manuscript. FL, TH, JS, JY, XX, JB, JM, MZ participated in algorithm development and data analysis. All authors read and approved the final manuscript.

## Declarations

The publication of this article has been funded by NIH U54CA149169.

This article has been published as part of *BMC Systems Biology *Volume 7 Supplement 2, 2013: Selected articles from The 6^th ^International Conference of Computational Biology. The full contents of the supplement are available online at http://www.biomedcentral.com/bmcsystbiol/supplements/7/S2.

## References

[B1] Gupta PB Chaffer CL Weinberg RA Cancer stem cells: mirage or reality? Nat Med 2009 15 9 1010 1012 10.1038/nm0909-1010 19734877

[B2] Nguyen LV Vanner R Dirks P Eaves CJ Cancer stem cells: an evolving concept Nat Rev Cancer 2012 12 2 133 143 22237392 10.1038/nrc3184

[B3] Bonnet D Dick JE Human acute myeloid leukemia is organized as a hierarchy that originates from a primitive hematopoietic cell Nat Med 1997 3 7 730 737 10.1038/nm0797-730 9212098

[B4] Al-Hajj M Wicha MS Benito-Hernandez A Morrison SJ Clarke MF Prospective identification of tumorigenic breast cancer cells Proceedings of the National Academy of Sciences 2003 100 7 3983 3988 10.1073/pnas.0530291100 PMC153034 12629218

[B5] O'Brien CA Pollett A Gallinger S Dick JE A human colon cancer cell capable of initiating tumour growth in immunodeficient mice Nature 2007 445 7123 106 110 10.1038/nature05372 17122772

[B6] Pardal R Clarke MF Morrison SJ Applying the principles of stem-cell biology to cancer Nat Rev Cancer 2003 3 12 895 902 10.1038/nrc1232 14737120

[B7] Huntly BJ Gilliland DG Cancer biology: summing up cancer stem cells Nature 2005 435 7046 1169 1170 10.1038/4351169a 15988505

[B8] Rich JN Cancer stem cells in radiation resistance Cancer Res 2007 67 19 8980 8984 10.1158/0008-5472.CAN-07-0895 17908997

[B9] Zhang M Atkinson RL Rosen JM Selective targeting of radiation-resistant tumor-initiating cells Proc Natl Acad Sci USA 2010 107 8 3522 3527 10.1073/pnas.0910179107 20133717 PMC2840501

[B10] Horst D Chen J Morikawa T Ogino S Kirchner T Shivdasani RA Differential WNT activity in colorectal cancer confers limited tumorigenic potential and is regulated by MAPK signaling Cancer Res 2012 72 6 1547 1556 10.1158/0008-5472.CAN-11-3222 22318865 PMC3571091

[B11] Vermeulen L De Sousa EMF van der Heijden M Cameron K de Jong JH Borovski T Tuynman JB Todaro M Merz C Rodermond H Wnt activity defines colon cancer stem cells and is regulated by the microenvironment Nat Cell Biol 2010 12 5 468 476 10.1038/ncb2048 20418870

[B12] Anderson AR A hybrid mathematical model of solid tumour invasion: the importance of cell adhesion Math Med Biol 2005 22 2 163 186 10.1093/imammb/dqi005 15781426

[B13] Kansal AR Torquato S Harsh GI Chiocca EA Deisboeck TS Simulated brain tumor growth dynamics using a three-dimensional cellular automaton J Theor Biol 2000 203 4 367 382 10.1006/jtbi.2000.2000 10736214

[B14] Bauer AL Jackson TL Jiang Y A cell-based model exhibiting branching and anastomosis during tumor-induced angiogenesis Biophys J 2007 92 9 3105 3121 10.1529/biophysj.106.101501 17277180 PMC1852370

[B15] Frieboes HB Zheng X Sun CH Tromberg B Gatenby R Cristini V An integrated computational/experimental model of tumor invasion Cancer Res 2006 66 3 1597 1604 10.1158/0008-5472.CAN-05-3166 16452218

[B16] Frieboes HB Lowengrub JS Wise S Zheng X Macklin P Bearer EL Cristini V Computer simulation of glioma growth and morphology Neuroimage 2007 37 Suppl 1 S59 70 17475515 10.1016/j.neuroimage.2007.03.008 PMC2243223

[B17] Zhu X Zhou X Lewis MT Xia L Wong S Cancer stem cell, niche and EGFR decide tumor development and treatment response: A bio-computational simulation study J Theor Biol 2011 269 1 138 149 10.1016/j.jtbi.2010.10.016 20969880 PMC3153880

[B18] Ganguly R Puri IK Mathematical model for the cancer stem cell hypothesis Cell Prolif 2006 39 1 3 14 10.1111/j.1365-2184.2006.00369.x 16426418 PMC6495990

[B19] Michor F Mathematical models of cancer stem cells J Clin Oncol 2008 26 17 2854 2861 10.1200/JCO.2007.15.2421 18539964

[B20] Sottoriva A Verhoeff JJC Borovski T McWeeney SK Naumov L Medema JP Sloot PMA Vermeulen L Cancer Stem Cell Tumor Model Reveals Invasive Morphology and Increased Phenotypical Heterogeneity Cancer Research 2010 70 1 46 56 10.1158/0008-5472.CAN-09-3663 20048071

[B21] Tan H Li F Singh J Xia X Cridebring D Yang J Zhan M Wong S Bao J Ma J A 3-dimentional multiscale model to simulate tumor progression in response to interactions between cancer stem cells and tumor microenvironmental factors 2012 IEEE 6th International Conference on Systems Biology (ISB) 18-20 Aug. 2012 2012 2012 297 303

[B22] Hori K Suzuki M Abe I Saito S Increased tumor tissue pressure in association with the growth of rat tumors Jpn J Cancer Res 1986 77 1 65 73 3082817

[B23] Morrison SJ Kimble J Asymmetric and symmetric stem-cell divisions in development and cancer Nature 2006 441 7097 1068 1074 10.1038/nature04956 16810241

[B24] Dingli D Traulsen A Michor F (A)symmetric stem cell replication and cancer PLoS Comput Biol 2007 3 3 e53 10.1371/journal.pcbi.0030053 17367205 PMC1828703

[B25] Zheng X Wise SM Cristini V Nonlinear simulation of tumor necrosis, neo-vascularization and tissue invasion via an adaptive finite-element/level-set method Bull Math Biol 2005 67 2 211 259 10.1016/j.bulm.2004.08.001 15710180

[B26] Macklin P McDougall S Anderson AR Chaplain MA Cristini V Lowengrub J Multiscale modelling and nonlinear simulation of vascular tumour growth J Math Biol 2009 58 4-5 765 798 10.1007/s00285-008-0216-9 18781303 PMC3037282

[B27] Truskey G Yuan F Katz D Transport Phenomena in Biological Systems Pearson Prentice Hall, Upper Saddle River, NJ 2004

[B28] Gockenbach MS Understanding and implementing the finite element method Philadelphia: Society for Industrial and Applied Mathematics 2006

[B29] Shu SGaC Total variation diminishing runge-kutta schemes Mathematics of computation 1998 67 73 85 10.1090/S0025-5718-98-00913-2

[B30] Weissman IL Stem cells: units of development, units of regeneration, and units in evolution Cell 2000 100 1 157 168 10.1016/S0092-8674(00)81692-X 10647940

[B31] Norton L A Gompertzian model of human breast cancer growth Cancer Res 1988 48 24 Pt 1 7067 7071 3191483

[B32] Paez-Ribes M Allen E Hudock J Takeda T Okuyama H Vinals F Inoue M Bergers G Hanahan D Casanovas O Antiangiogenic therapy elicits malignant progression of tumors to increased local invasion and distant metastasis Cancer Cell 2009 15 3 220 231 10.1016/j.ccr.2009.01.027 19249680 PMC2874829

[B33] Ebos JM Lee CR Cruz-Munoz W Bjarnason GA Christensen JG Kerbel RS Accelerated metastasis after short-term treatment with a potent inhibitor of tumor angiogenesis Cancer Cell 2009 15 3 232 239 10.1016/j.ccr.2009.01.021 19249681 PMC4540346

